# Extracellular Vesicles and Matrix Remodeling Enzymes: The Emerging Roles in Extracellular Matrix Remodeling, Progression of Diseases and Tissue Repair

**DOI:** 10.3390/cells7100167

**Published:** 2018-10-13

**Authors:** Muhammad Nawaz, Neelam Shah, Bruna Riedo Zanetti, Marco Maugeri, Renata Nacasaki Silvestre, Farah Fatima, Luciano Neder, Hadi Valadi

**Affiliations:** 1 Department of Rheumatology and Inflammation Research, Sahlgresnka Academy, University of Gothenburg, Gothenburg 41346, Sweden; marco.maugeri@rheuma.gu.se (M.M.); hadi.valadi@gu.se (H.V.); 2 Department of Biochemistry and Molecular Biology, Monash University, Victoria 3800, Australia; neelam.shah85@yahoo.com; 3 Department of Pathology and Forensic Medicine, Ribeirao Preto School of Medicine, University of Sao Paulo, Ribeirao Preto 14049-900, Brazil; brunabrz@hotmail.com (B.R.Z.); renata.nacasaki@gmail.com (R.N.S.); farah.fatima00@gmail.com (F.F.); neder@fmrp.usp.br (L.N.)

**Keywords:** extracellular vesicles, exosomes, matrix metalloproteinases, MMPs, extracellular matrix, telocytes, tumor progression, cardiovascular diseases, arthritis, tissue repair

## Abstract

Extracellular vesicles (EVs) are membrane enclosed micro- and nano-sized vesicles that are secreted from almost every species, ranging from prokaryotes to eukaryotes, and from almost every cell type studied so far. EVs contain repertoire of bioactive molecules such as proteins (including enzymes and transcriptional factors), lipids, carbohydrates and nucleic acids including DNA, coding and non-coding RNAs. The secreted EVs are taken up by neighboring cells where they release their content in recipient cells, or can sail through body fluids to reach distant organs. Since EVs transport bioactive cargo between cells, they have emerged as novel mediators of extra- and intercellular activities in local microenvironment and inter-organ communications distantly. Herein, we review the activities of EV-associated matrix-remodeling enzymes such as matrix metalloproteinases, heparanases, hyaluronidases, aggrecanases, and their regulators such as extracellular matrix metalloproteinase inducers and tissue inhibitors of metalloproteinases as novel means of matrix remodeling in physiological and pathological conditions. We discuss how such EVs act as novel mediators of extracellular matrix degradation to prepare a permissive environment for various pathological conditions such as cancer, cardiovascular diseases, arthritis and metabolic diseases. Additionally, the roles of EV-mediated matrix remodeling in tissue repair and their potential applications as organ therapies have been reviewed. Collectively, this knowledge could benefit the development of new approaches for tissue engineering.

## 1. Introduction

Extracellular matrix (ECM) provides mechanical and chemical support to cells and orchestrates the structural framework and homeostasis of connective tissues where the local components and composition of ECM collectively determine the biochemical properties of the connective tissue [1,2,3]. The composition of ECM varies among multicellular structures whereby fibroblasts and epithelial cells are the most common cell types in the stromal architecture. Additionally, the collagen, proteoglycans, elastin, fibronectin and integrins are also important parts of the interstitial architecture. Unlike fibroblasts, the polarized organization of epithelial cells is strengthened by cell-to-cell and cell-to-ECM interactions through adhesion proteins or re-localization of certain proteins to appropriate domains of the plasma membrane [4,5]. Other components which facilitate cell-to-cell and cell-to-ECM communications within the given tissue niche, include adhesion molecules (CAMs i.e. integrins), fibronectins and surface receptors. Intercellular communication via fibronectin leads to cell-dependent changes in the ECM of a certain tissue [6].

The interstitial Cajal-like cells (ICLC), known as telocytes (TCs), have been lately reported as a population of the connective tissue, which are known to make cellular junctions [7,8,9], and have been implicated in variety of human pathological states [10]. TCs are thought to serve as connecting devices, largely due to their ability to interact with themselves through homocellular junctions as well as with other cell types (for details see [11]). Not only with cells, TCs could also establish physical contacts with surrounding structures such as blood vessels, nerve endings, smooth muscles, glandular elements, and epithelial coverings [9,12,13]. In addition to physical contacts, the acellular or paracrine components of ECM, which include cell-derived secretome such as secreted soluble proteins, growth factors, cytokines and chemokines, facilitate the paracrine functional coordination in a structural framework of a connective tissue.

Importantly, the cellular interactions and communication could also be facilitated by direct contact between cells such as cellular gap junctions and the tuning nanotubules (TNTs) [14], and by recently discovered secreted vesicles named extracellular vesicles (EVs). EVs are nano- and micro-sized membrane enclosed vesicles secreted constitutively or actively by almost every cell type and are packaged with components of their cellular origin. The process of EVs secretion seems to be ubiquitous i.e. secreted under both physiological and pathological conditions, and appears to be evolutionarily conserved across the species’ cross-kingdoms [15], ranging from prokaryotes to eukaryotes. Based on their mode of biogenesis, origin, size, and sedimentation densities, EVs are categorized as heterogeneous population of vesicles [16]. The well characterized are the exosomes and microvesicles, which differ in their origin, size and densities. Exosomes originate from endosomes whereas microvesicles are produced from outward budding of the plasma membrane, also designated as membrane-derived vesicles or shedding vesicles. Recently, Dolores Di Vizio group has reported the large sized membrane-derived vesicles known as large oncosomes (1–10 μm diameters) as a discrete class of EVs [17,18,19].

The biogenesis, maturation of various populations of EVs, sorting of molecules and their secretion into extracellular milieu is regulated by several factors (for detailed mechanisms see [20,21]). Exosomes are formed as intraluminal vesicles within the lumen of endosomes and are matured in multivesicular bodies (MVBs), which upon fusion with the plasma membrane are secreted into the extracellular milieu. In contrast, the microvesicles are produced (bud off) from the plasma membrane and are directly secreted outside. EVs carry a repertoire of biological molecules such as RNAs including mRNAs [22,23], miRNAs and ribosomal RNAs [23], and long non-coding RNAs (lncRNAs) [24]. EVs also contain DNA including genomic and mitochondrial DNA (mtDNA) and proteins, lipids and carbohydrates among others. The detection of these secreted molecules in circulating EVs obtained from various body fluids makes them an ideal source of disease biomarkers [25,26].

One of the most profound characteristics of EVs that have been widely reported is their participation in intercellular communication by transporting molecules and transmitting biological messages between cells and play physiological roles [27]. Due to their abilities of transporting bioactive molecules and pathological signals, EVs actively participate in the progression of various diseases. For instance, EV-mediated spread of misfolded and toxic proteins and specific miRNAs has been implicated in neurodegenerative diseases [28,29], and other physio-/pathological functions of the brain [30,31]. Additionally, the contribution of EVs in the dissemination of oncogenic content such as the aberrantly expressed genes, nucleic acids and oncogenic proteins plays active roles in cancer biology [32,33,34,35,36]. Importantly, EVs play active roles in tissue regenerative processes and ameliorate the organ functions by potentially stimulating the repair processes. EV-mediated transport of bioactive molecules to the sites of injury has been attributed for stimulating and regulating intrinsic regenerative programs in damaged tissues [37,38].

The augmented body of evidence has suggested that intercellular interactions could be disrupted physically and functionally by EV-mediated proteolytic activities, as EVs contain matrix-degrading enzymes such as matrix metalloproteinases (MMPs), heparanases, hyaluronidases, and extracellular matrix metalloproteinase inducer (EMMPRIN), and aggrecanases such as adamalysin metalloproteinases having disintegrin and thrombospondin domains (ADAMTSs) and tissue inhibitors of metalloproteinases (TIMPs), among others. As such, the presence of proteolytic molecules in EVs is one of the newly discovered sources for tissue remodeling through pericellular gelatinolytic activities (proteolysis), which modulate the structural architecture and dynamics of ECM and contribute for both the development of various diseases, tissue regeneration and amelioration of organ functions that will be discussed in this review.

## 2. MMPs Secretion into EVs

The proteases in EVs from different sources are secreted in a context dependent manner [39]. For their conversion into functionally active MMPs, the membrane type MMPs are internalized to the endosomes and are recycled to the plasma membrane [40]. However, recent reports demonstrate that instead of recycling, the membrane type proteins from endosomes could be cleaved, converted into functionally active soluble forms and packaged into the intraluminal vesicles and secreted via EVs [41]. Membrane type 1 matrix metalloproteinase (MT1-MMP), also known as MMP14 belongs to transmembrane family of MMPs, but surprisingly, it was recently detected in cell culture media as a content of EVs [32,42,43]. MMP14, after its internalization to the endosomes was not recycled back to the plasma membrane, but was packaged into EVs and secreted into the extracellular environment and was shown to be functionally active soluble form [42].

Although, the reports have demonstrated possible mechanisms by which miRNAs and proteins (other than MMPs) are regulated for their secretion into EVs [44,45,46], nevertheless, the mechanisms regarding the secretion of MMPs into EVs remain largely unknown. Recently, it was shown that the stabilization of MMPs in MVBs and their secretion into EVs is regulated by cortactin [47]. This is in agreement with previous reports showing that cortactin regulates the trafficking and secretion of MMPs [48,49]. Importantly, a specific subset of Rab GTPases such as Rab5a, Rab8a and Rab14 are crucial for regulating cell surface presentation of MT1-MMP complex [50,51]. Rab GTPases are co-localized with MT1-MMP positive vesicles, indicating their involvement in trafficking of MT1-MMP vesicles for surface presentation of MMPs. In addition to the Rab GTPases, the kinesins could also drive cell surface exposure of MT1-MMP and promote CD44 shedding in primary macrophages [51]. Masci and colleagues propose that EVs dispersed along the fibroblast cell periphery contain abundance of MMPs [52]. The authors also demonstrate that the fusion of intraluminal vesicles with the outer membrane microvesicles (MVs) could act as a controlling mechanism during which MMP-9 is first activated (i.e., cleaved) and then released extracellularly. Recently, Clancy and colleagues have reported that v-SNARE and VAMP3 regulate the delivery of vesicle cargo such as the MT1-MMP (MMP14) to shedding microvesicles [53].

It is important to consider that the production of MMPs and their activities are largely influenced by MMP inducers and regulators such as EMMPRIN and TIMPs respectively. For instance, human uterine epithelial cells secrete EMMPRIN in EVs, which interact with recipient human uterine fibroblasts (HUFs) or uterine epithelial cells and stimulates the production of MMPs in these recipient cells. Such release of EMMPRIN-containing EVs from HUF cells is accelerated by stimulation of G protein-coupled receptor 30 [54]. Additionally, the TIMPs have been detected in EVs in the form of complex with MMPs i.e., TIMP-MMP complexes in EVs, such as those released by trabecular meshwork (TM) cells [55]. However, in a previous study TIMP-2 detected in EVs from TM cell cultures was not in the form of complex with MMPs [56].

What comes more is that MMP-mediated cell surface processing could generate functionally active soluble form of transmembrane proteins such as epithelial cell adhesion molecule (EpCAM), TNFR1 and CD46 [57]. Likewise, serum MMPs could contribute to proteolytic cleavage of EpCAM, which is packaged into ascites-derived EVs of ovarian and breast cancer patients [58]. In addition to cell surface cleavage, the proteolytic processing can also occur within endosomal compartments such as MVBs. For instance, the adhesion molecules such as L1 (CD171) and CD44 molecules are cleaved in the endosomal compartment of cells and are released with EVs as soluble forms [59]. EVs also contribute to redistribution of CD10 peptidase activity from cell surface to extracellular microenvironment, which takes place by tetraspanin protein CD9-mediated release of metalloprotease CD10 [60].

Interestingly, EVs share homologous structures to those with matrix vesicles (MVs), which are characteristic of matrix mineralization (mineralization of calcified cartilage, bone and dentin) and are secreted from plasma membranes of differentiating growth plate chondrocytes, odontoblasts and osteoblasts. Recently, Karin and colleagues have shown that EVs of late stage of osteogenic differentiation could induce ECM mineralization [61]. However, with regard to matrix MVs, the coordinated signaling response observed in chondrocyte-derived MVs and the mineralization of vascular smooth muscle cell-derived MVs is considered a pathological response against deregulated intracellular calcium homeostasis [62]. These MVs contain matrix-processing enzymes such as MMP-2, MMP-9 and MMP-13 that contribute important role in matrix remodeling mainly through degradation of proteoglycans and allow calcification [63,64].

Aikawa and colleagues argue that although, much as with MVs, the calcifying EVs in the fibrillar collagen ECM of cardiovascular tissues serve as calcification foci, nevertheless the formed mineral appears different between the tissues [65]. Additional studies have suggested that the activation of TGF-β takes place through MVs containing MMP-3, which are produced by growth plate chondrocytes [66,67], and that the production of MVs is induced by phosphate through extracellular signal-regulated kinases Erk1/2 pathway. Hitherto, MV and EVs exhibit similar activities for mineralization of ECM, but are biological distinct entities [65,68].

**Figure 1:**
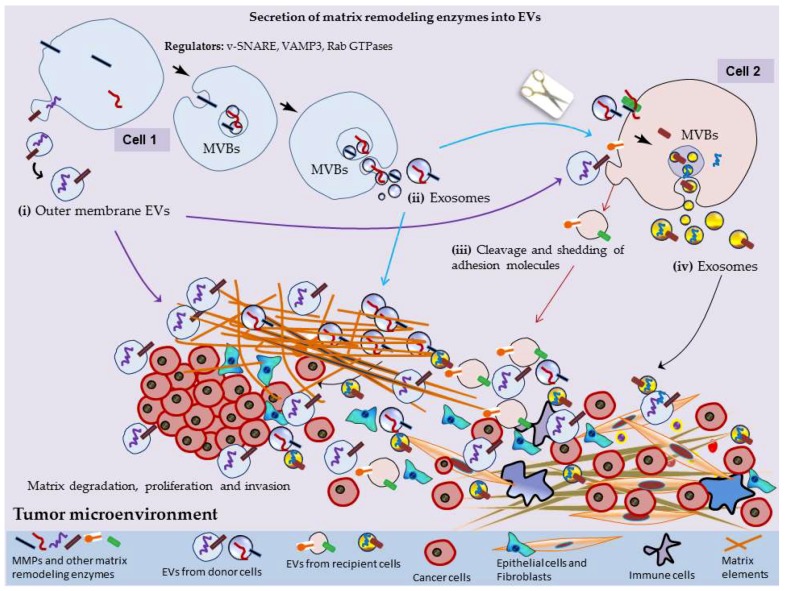
Schematic illustration of secretion of extracellular vesicles (EV)-associated matrix degrading enzymes, matrix remodeling and tumor progression: EVs (both outer membrane microvesicles and endosome-derived exosomes: (i) and (ii) respectively) secreted from cells carry plethora of matrix degrading enzymes on their surface or packed inside the lumen of EVs. EV-associated matrix cleaving enzymes are secreted along the entire cell periphery and can interact with neighboring cells (cell 2) whereby they cleave the surface proteins, transmembrane and adhesion molecules of recipient cells, which are either packaged into the outer membrane vesicles of recipient cells or shed directly into cells’ periphery (iii). Additionally, EVs taken up by recipient cells can induce further production of matrix metalloproteinases (MMPs) and their release via EVs of recipient cells (iv). Such activities occurring along the cell periphery play active roles in matrix degradation and facilitate cellular invasion, metastasis and preparation of tumor permissive niche. MVBs: multivesicular bodies.

What comes more important is that when EVs interact with other cells, the MMPs present on the surface of EVs promote the cleavage of the recipient cells’ surface proteins, transmembrane proteins and adhesion molecules. Such surface cleavage allows either direct shedding or subsequent packaging of cleaved surface proteins into outer membrane-derived EVs (microvesicles) of these cells [69,70,71,72,73]. Buzas and colleagues have provided an overview of known components of EV surface interactome, explaining that some of the EV surface molecules are shared with those of the plasma membrane of the releasing cell and have highlighted some of the already established roles of EV surface interactions in different biological processes [74]. The secretion and high number of EVs in biological fluids, represent a uniquely large interactive surface area, which can establish contacts both with cells and with molecules in the extracellular microenvironment [74] In this context, the presence of matrix processing enzymes in EVs and their secretion along the entire cell periphery (Figure 1) provides an additional layer of interactome and play active role in pathological and physiological processes, which has been discussed in the sections below.

## 3. EV-Mediated Surface Interactions, Matrix Remodeling and Tumor Progression

The MMPs presented on the surface of EVs appear to derive diverse proteolytic activities for ECM turnover, and localized degradation of ECM, and thus contribute to matrix remodeling [41,75,76,77]. As such, the aberrant deposition or the loss of ECM components could bring numerous changes in the microenvironment, which is occasionally associated with the development of diseases. Therefore, being rich in proteases, the tumor-derived EVs could potentially modulate the ECM in a way, which favors tumor progression (Figure 1). A variety of MMPs and MMP regulators (i.e., EMMPRINs and TIMPs) is secreted into extracellular environment via EVs and could play active roles in modulating matrix biology. The most frequently detected ones are listed in Table 1.

The selective localization of MMPs such as MMP-9 and β-1 integrin and their shedding into EVs from tumor cells participate in localized degradation and the proteolysis of ECM during cellular migration and metastasis [86]. The presence of integrins such as CD41, at the surface of cancer cells enhances the adhesion of cancer cells with endothelial cells. As such, EVs secreted from platelets transfer platelet-derived integrin CD41 to the surface of breast cancer cells and enhance the invasive properties of breast cancer cells [33]. Furthermore, tumor-derived EVs could induce the expression of MMPs in recipient cells. For example, EV-associated heat shock protein-90 released by invasive cancer cells induce the expression of MMP-2 which activates plasmin, a second protease that promotes cancer cell invasion [87]. It has been shown that EVs shed by tumor cells may facilitate the activation of membrane-associated proteinases, particularly those involved in matrix degradation and tissue invasion [86]. In fact, EVs shed by tumor cells contain the most active forms of aggrecanases such as ADAMTSs including Adamts1, Adamts4 and Adamts5, which elicit elevated proteolytic activity [103]. Generally, these aggrecanases exhibit proteolytic activities at the sites previously identified as targets for ADAMTSs [107].

Head and neck squamous cell carcinoma-derived EVs have been shown to modulate the expression of matrix remodeling proteins e.g. EFEMP1, DDK3, SPAR [108]. MMP-13 that reportedly has been shown to be overexpressed in various tumors and is associated with increased risk of metastasis in patients with head and neck cancer [109,110], was recently detected in nasopharyngeal cancer (NPC) patient plasma-derived EVs [94]. It is believed that the overexpression of hypoxia-induced MMP-13 in NPC-derived EVs can induce changes in tumor microenvironment whereas MMP-13 levels are strongly associated with lymph node metastasis, clinical stage and poor prognosis in NPC patients [97]. This study also shows that EV-associated MMP-13 up-regulates the Vimentin expression while decreasing E-cadherin levels in NPC cells in vitro and in vivo. Additional studies have demonstrated that the degradation of collagens and fibronectin facilitate cell adhesion and motility, invasion and angiogenesis, and promote clotting after vessel disruption [111]. Similarly, the fibronectin-assisted adhesion of EVs to myeloma cells stimulates the expression of MMP-9 in recipient cells, which promotes the myeloma progression [112].

In melanoma cells, microtubule-dependent traffic of MMP-2 and MMP-9 containing vesicles and their exocytosis promote cancer cell invasion [113]. Of note, anti-myeloma chemotherapy stimulates the elevated secretion of EVs containing high levels of heparanase on their surface, which degrade ECM and can be transferred to both tumor and host cells, altering their behavior in a way that enhances tumor survival and progression [114]. The presence of hyaluronidase Hyal1 in prostate cancer-derived EVs stimulate the prostate stromal cell motility by engaging FAK-mediated integrin signaling, indicating that the elevated Hyal1 promotes prostate cancer progression [115]. Collectively, the disruptions in cellular adhesion and interactions promote tumor growth by facilitating cell motility, proliferation and tumor cell invasion, and are strongly correlated with poor prognosis and shorter overall survival [116,117,118,119]. The following sections will discuss the mechanisms by which EV-associated MMPs influence the tumor progression.

### 3.1. Construction of Pre-Metastatic Niche

The components and activities of ECM define the biophysical architect of pre-metastatic niche. It is well known that the localized degradation of ECM may educate cells to move to anatomically new location where they interact with normal resident cells and educate them towards tumor cell phenotypes. The role of EVs in priming pre-metastatic niche was first described by Peinado et al. showing that melanoma-derived EVs educate the bone marrow progenitor cells toward a pro-metastatic phenotype through mesenchymal transition [120]. EVs play active roles in pre-metastatic niche formation where tumor cells benefit from the secretion of EVs as they influence the behavior of neighboring tumor cells within the tumor microenvironment [120,121,122,123,124]. Importantly, tumor-derived EVs not only influence the local microenvironment but also play active role in distal metastatic dissemination and allow the metastatic organotropism of different tumor types [125,126,127].

It has been shown that homing of melanoma-derived EVs to sentinel lymph nodes have a role in priming the lymph node environment, which stimulates proangiogenic program through ECM deposition in the lymph nodes [128]. Thus, EV-mediated process of micro-anatomic niche preparation facilitates the lymphatic metastasis. The preparation of pre-metastatic niche at new anatomical site requires the recruitment of the cancer cells, immune cells, growth factors, and MMPs. Keeping in view the widely accepted roles of MMPs in the preparation of pre-metastatic niche [129,130]; EV-associated MMPs could therefore be potential biological entities attributed in matrix remodeling and the construction of pre-metastatic niche.

MMPs’ activity results in the loss of cellular adhesion and increased motility, which collectively facilitate homing of tumor cells to new location. Tumor cell-derived EVs are potent stimulators of MMP-9, IL-6, and TGF-β1 and induce the secretion of extracellular EMMPRIN, which play active roles in driving immune evasion, invasion and inflammation in the tumor microenvironment [131]. It has been shown that hrombospondin-1 and its receptor CD47 are expressed on T cell-derived EVs and are internalized by both T cells and ECs and modulate signaling in recipient cells [132].

Additional studies demonstrate that tumor-derived EVs could influence the breast cancer cells’ invasion through differential glycosylation of EMMPRIN, associated with p38/MAPK signaling pathway, which is activated by EMMPRIN-containing EVs [80]. It has been reported that CD105-positive EVs contribute the establishing of pre-metastatic niche in the lung microenvironment of SCID mice by upregulating MMP-2, MMP-9 and VEGFR1 [133].

### 3.2. Fibroblastic Switching and Acquisition of Mesenchymal Mode

ECM-associated anomalies may affect cancer progression by promoting cellular transitions. It is well known that matrix degradation minimizes the adherent properties of cancer cells in order to promote their motility and invasion. As, such the motile cells may acquire the elongated forms, and assume novel phenotypes such as mesenchymal mode and the acquisition of fibroblast-like phenotypes that could be associated with distinct amoeboid forms and the shedding of large EVs (i.e., large oncosomes) into the extracellular space [18,134,135]. Several studies have highlighted that the potency of cancer cell migration and invasion is largely dependent on the acquisition of mesenchymal cell state. A well demonstrated example is epithelial-to-mesenchymal transition (EMT); often characterized by the secretion of MMPs, weak intercellular adhesion and reduced cellular polarity which is implicated in metastasis and acquisition of malignant traits [136,137,138,139,140,141].

Several studies have clarified that EVs mediate and facilitate the phenotypic switching of cells towards mesenchymal transitions including both EMT and MET [120,142,143,144,145,146,147]. EVs secreted from one cell type are taken up by other cell type, which could induce EMT-like process in recipient cells [148]. Breast cancer-derived EVs stimulate myofibroblast differentiation and impose consequential changes in extracellular matrix and the vascular sprouting in adipose stem cells. This incites desmoplastic reprogramming in the tumor microenvironment and tumor cells’ glutamine metabolism [149]. TIMP-deficient fibroblasts could lose the metalloproteinase activity within the tumor-stromal compartment, demonstrating that the loss of TIMPs promotes the acquisition of cancer associated fibroblasts (CAFs) characteristics [101]. Since, TIMPs are major regulators (inhibitors) of the proteolytic activities of MMPs, TIMP deregulation may result in cancer progression [101,150].

### 3.3. Tumor Neovascularization

Augmented body of evidence has revealed the participation of EV-associated growth factors in angiogenesis however; the role of EV-associated MMPs in neo-angiogenesis has only more recently started being explored. Since endothelial cells (ECs) are more active for vascularization, it has been shown that endothelial progenitor cell-derived EVs activate angiogenic programs in ECs through transferring mRNA [151]. This is in agreement with the study by Ratajczak et al. which for the first time showed that EVs could transfer mRNA horizontally from one cell to the other cell and induce the phenotypic changes in recipient hematopoietic progenitor cells [22], followed by study from Deregibus et al. showing that the endothelial progenitor cell-derived EVs activate an angiogenic program in ECs by a horizontal transfer of mRNA.

Recently, it was shown that CD105^+^ EVs contain several mRNA transcripts of proangiogenic factors such as VEGF, FGF, angiopoietin1, ephrin A3, MMP-2, and MMP-9 and promote the angiogenic activities in vitro as well as in vivo [133]. Prostate cancer cell-derived EVs contain tumor growth factor (TGFβ) and stimulate the differentiation of bone-marrow mesenchymal stem cells (BM-MSCs) into myofibroblasts, which secrete high levels of VEGF-A, HGF and MMPs such as MMP-1, -3 and -13, have pro-angiogenic functions and enhanced tumor cell proliferation [98].

The CAFs secrete ADAM10-rich EVs that promote cell motility and activate RhoA and Notch signaling in cancer cells [101]. RhoA signaling is essential to facilitate ECs’ migration and VEGF-mediated angiogenesis [152,153]. It has been shown that the increase in TIMP-1 levels may upregulate miR-210 by induction of pro-tumorigenic PI3K/AKT/HIF-1 signaling pathway, whereas EVs released by TIMP-1 overexpressing cells may exhibit significantly increased pro-angiogenic activities during lung cancer progression [104]. Initial studies by Ratajczak and colleagues showed that EVs released from platelet alpha-granules contain integrin CD41 and MT1-MMP that contribute in metastatic spread and angiogenesis in lung cancer [32].

CD147 (EMMPRIN) containing EVs secreted from epithelial ovarian cancer cells may stimulate proangiogenic activities in ECs through CD147-mediated MMP regulatory networks [83]. Importantly, the annexin A2 acts as a vehicle for the trafficking of CD147-positive EVs during tumor-stromal interactions and govern the production of MMP-2 from fibroblasts, which enhance the migration potential of cancer cells [90]. In fact, tumor and ECs secrete MMP-containing EVs, which regulate the localized proteolytic activity to generate permissive microenvironment during tumor angiogenesis [84]. Because increased matrix density results in decreased angiogenesis [154], therefore MMP-mediated proteolysis activities are required to regulate the neovasculature in microenvironment.

## 4. EV-Associated MMPs as Cancer Biomarkers

It has been shown that the concentrations of proteolytic content (i.e., MMPs) in human cancer cell-derived EVs are directly correlated with proteolysis-facilitated cell invasion [155]. Importantly, MMP-containing EVs from benign serous cysts confer minimal proteolytic activity as compared to malignant forms [155]. As such, the process of EVs’ release and the MMP-derived gelatinolytic activities differ at various stages of cancer, thus differentiating early stage cancer from the late stage, and benign tumors from the malignant ones [155,156,157]. Similarly, EVs from non-small cell lung-carcinoma (NSCLC) patients consistently showed significant increases of ADAM10sa compared to their normal controls and benign-tumors. Moreover, stage IA-IIB NSCLC primary tumors of patients who died of the disease exhibited greater increases of ADAM10sa compared to those of patients who survived 5 years following diagnosis and surgery [158]. This indicates that enzyme-specific proteolytic activities could be harvested as potential cancer biomarkers for early detection and outcome prediction. Additionally, multiple carcinoembryonic antigen-related cell adhesion molecules (CEACAMs) and ECM proteins were identified in EVs isolated from pancreatic duct fluid of pancreatic ductal adenocarcinoma (PDAC) patients [159], indicating their role in carcinogenesis of PDAC and diagnostic potential.

It has been reported that EVs isolated from ascites and sera of ovarian carcinoma patients containing MMP-2, MMP-9 and plasmin are capable of promoting proteinase-dependent metastatic activity in malignant ovarian epithelium, indicating their diagnostic value [92]. In addition to their MMP content, EVs from cancer cells may also contain high amounts of claudin proteins, which are known to facilitate cancer progression. Importantly, EVs derived from ovarian cancer patients positive for claudin-4 are also positive for CA125 [160]. It is interesting to consider that claudin-4 and CA125 could be tested in combinations for their possible use as prognostic biomarkers in ovarian cancers [161]. Several EV-associated claudins and adhesion molecules in ovarian cancer are reviewed elsewhere [161].

## 5. EV-Driven Matrix Destruction and Development of Arthritic Diseases

Several studies have demonstrated that EVs play prominent roles in the pathophysiology of arthritic diseases including osteoarthritis (OA) and rheumatoid arthritis (RA) [95,162,163,164,165]. RA models have shown that EVs stimulated with inflammatory cytokines could induce extracellular damage facilitated by matrix-degrading enzymes. The major processes by which these membrane-bound vesicles and their associated MMPs destruct the joint tissue include ECM degradation, invasion and the modulation of inflammation.

The platelet-derived EVs promote migration, invasion and adhesion in ECM of RA fibroblast-like synoviocytes (FLSs), which play important roles in the progression of joint destruction. EVs could up-regulate the expression of MMP1 accompanied by increased levels of phosphorylation of p-NF-κB and p-Erk in RA-FLSs [166]. This indicates that EVs promote RA-FLSs adhesion and motility by increasing MMP-1 via activating Erk-mediated NF-κB pathway. Additionally, EVs isolated from endothelial cells carry MMP-2, MMP-9, and MT1 indicating the involvement of EVs in the breakdown of capillary membrane [79], which could lead to fluid build-up, swelling, and homing of cells and proteins from the joint space into the systemic circulation [95].

MMPs break down the proteoglycans, such as aggrecan and collagen in the ECM and are thought to be a major cause of cartilage destruction in RA. In this context, synovial tissue-derived EVs breakdown the cartilage tissue and result in joint destruction. For example, EVs shed by FLSs contain ADAMTS-5, which degrade aggrecan and facilitate oligodendroglioma and RA-FLSs to invade through aggrecan-rich ECM [103]. EVs released by synovial fibroblasts contain hexosaminidase D and B-glucuronidase enzymes, which show enzymatic activity similar to aggrecanase in the joint space of patients with both RA and OA. Hexosaminidase D is elevated in synovial fluid EVs of both patients with RA and OA and exhibit elevated hexosaminidase activity [167,168]. Previously, B-glucuronidase was thought to be a housekeeping gene but its presence in EVs from both RA and OA patients indicates that it is involved in regulation of ECM turnover, which represents yet another catabolic process in the development of OA and RA through EVs [95].

Additionally, EVs secreted from IL-1β-stimulated FLSs could trigger the up-regulation of MMP-13 and ADAMTS-5 expression in the articular chondrocytes and induce osteoarthritic changes both in vitro and in ex vivo models [93]. By contrast, adipose-derived EVs are capable to inhibit MMPs activity and have protective effects in OA chondrocytes [96,169], and can be potentially useful for new therapeutic approaches in joint repair (reviewed elsewhere [170]).

## 6. EV-Associated MMPs: Roles in Cardiovascular Diseases

The roles of EVs have been extensively described for cardiovascular diseases and atherosclerosis [171,172,173,174,175,176,177,178]. However, the contribution of EVs in cardiovascular diseases through matrix degradation is scarce in the literature and this area is gaining recent focus of interest.

It has been shown that during acute myocardial infarction (MI), there is an increase of platelet-derived EVs, EC-derived EVs and monocyte-derived EVs, which show strong association to atherosclerotic burden and risk of cardiovascular events. EVs were evaluated in whole blood from 105 patients with MI during a two-year follow-up [179]. Patients with non-ST-elevated MI had higher concentrations of CD41^+^ EVs, compared to ST-elevated MI patients. It was shown that CD62^+^ EVs counts were higher in MI patients with diabetes, and patients with hypertension had increased levels of CD14^+^ EVs, whereas the higher concentrations of CD62^+^ EVs early after MI were associated with an increased risk of cardiovascular events during follow up. The levels of platelet-derived EVs, EC-derived EVs and monocyte- derived EVs’ concentrations during long-term follow up after MI seem to reflect the underlying cardiovascular disease rather than the acute MI [179].

Additionally, the parvovirus B19-induced vascular damage occurring in the heart is associated with elevated circulating EC-derived EVs in human samples. However, the EC-derived EV subpopulation patterns were significantly different in B19V+ myocarditis in humans and transgenic B19V mice reflecting vascular damage [180]. This proposes that EC-derived EV profiles might permit differentiation between EC-mediated myocardial B19V infection and other causes of myocarditis [180]. EC-derived EVs interact with ECM and act as centers of MMP-2 activation and localization in order to incite vascular matrix remodeling [75]. EVs secreted by cardiomyocyte progenitor cells and MSCs are efficiently taken up by ECs, which stimulate EC migration and vessel formation. This effect is largely mediated via EVs containing EMMPRIN [181].

It is important to consider that the vascular damage is one of the major factors contributing in cardiac diseases, where EVs are thought to serve as essential mediators of cardiovascular calcification [182]—a major determinant of cardiovascular mortality [183]. In fact, vascular calcification leads to severe cardiovascular events in patients with hypertension, atherosclerosis, diabetes and chronic kidney disease for which matrix vesicles and EVs play important roles (see details [65,182]). The mineral imbalance in these pathological conditions can incite smooth muscle cells, valvular interstitial cells, and macrophages to release calcifying EVs containing specific mineralization-promoting cargo [65].

## 7. EV-Driven Matrix Remodeling: Metabolic Diseases and Others

EVs from visceral adipose tissue integrate into liver cells and cause the increased expression of TIMP-1, TIMP-4, and MMP-9 and decreased expression of MMP-7 and plasminogen activator inhibitor-1 in recipient cells. EV-induced expression of these molecules results into the dysregulation of TGF-β pathway in hepatocytes allowing the pathogenesis of nonalcoholic fatty (obesity-related) liver disease [76]. Additionally, it has been reported that vitamin D modulates the oxidative stress, inflammation, and EV biogenesis gene networks by dysregulating the expression of matrix extracellular phosphoglycoprotein, MMP-1 and MMP-28 genes (which are target of Runx2) in osteosarcoma cells [184].

Proteolytically active disintegrin and metalloproteinase (ADAMs) such as ADAM10 and ADAM17 carried by EVs are secreted during human abdominal aortic aneurysm (AAA) in smokers. The cultured neutrophils exposed to tobacco smoke extract showed increased ADAM10 and ADAM17 content, active cleavage of these molecules and release into EVs, which provides a novel molecular mechanism for the increased risk of AAA in smokers [99].

## 8. EV-Associated MMPs: Roles in Immunosuppression and Reproduction

The regulation of EV-associated proteolytic activities plays active roles in physiological processes such as reproduction [52]. The gelatinases such as MMP-2 and MMP-9 found in the seminal vesicles and ventral prostate of *Meriones libycus* are involved in the seasonal reproductive cycle. In fact, the immunosuppression of MMP-2 and MMP-9 in seminal vesicles has been observed during seasonal cycle of reproduction [52].

Recently, it was shown that the content of fibrillar collagens in seminal vesicles was elevated in hyperhomocysteinemic rats. Hyperhomocysteinemia increased the expression of MMP-2, -3, -7 and -9 in seminal vesicles [185]. The accumulation of collagen and upregulation of MMPs in seminal vesicles might contribute to the physiological remodeling of seminal vesicles. Additionally, in response to ovarian hormones, the MMP production from human uterine fibroblasts is regulated by secretion of intact EMMPRIN, proinflammatory cytokines and the activation of protein kinase C [82]. In addition, the presence of MMPs in EVs and their physio-/pathological functions have been reviewed elsewhere [186,187].

## 9. EV-Driven Matrix Remodeling: Roles in Tissue Repair and Therapies 

### 9.1. Joint Repair

EVs present in synovial fluid and cartilage ECM are involved in joint development and in the regulation of joint homeostasis [170]. The knowledge already acquired in this field suggests a role for EVs as biomarkers of joint disease, and as new tools to restore joint homeostasis and enhanced articular tissue regeneration offering new therapeutic approaches for joint repair [170]. It was shown that adipose MSC (adMSC)-derived EVs regulate MMPs activity and protect cartilage and bone degradation in OA [96].

The treatment of OA chondrocytes with human adMSC-EVs inhibits MMPs activity in chondrocytes and have protective effects in OA chondrocytes—raising their potential as new therapeutic approaches in damaged joint conditions [169]. Additionally, EVs exert a beneficial therapeutic effect on OA model by maintaining the balance between synthesis and degradation of chondrocyte (cartilage) ECM [188]. Monocyte-derived EVs stimulate cytokine secretion from MSCs, upregulate the expression of genes encoding for MMPs and facilitate tissue remodeling through EV-mediated signaling during the transition from injury and inflammation to bone regeneration and play an important role in the coupling between bone resorption and bone formation [189].

Besides proteins, several other molecules such as lipids, glycans, and nucleic acids are also players of EV surface interactions [74], and are also exported to the ECM, which regulate process of bone formation, inhibit osteoclast activity, and promote fracture repair [190]. Such EV-cargo could be utilized for molecular therapy in several skeletal disorders such as osteoporosis, osteogenesis imperfecta, and fracture healing. Collectively, EV-mediated signaling and ECM remodeling might represent an additional mode of activating cells’ intrinsic repair programs during the transition from injury to bone regeneration and inflammation resolve, thereby playing important role in the bone repair.

### 9.2. Corneal/ Ocular Repair

Ocular hypertension caused by ECM accumulation in the trabecular meshwork is a hallmark of glucocorticoid-induced glaucoma. As such, corticosteroid-induced alterations in adhesion cargo of EVs’ and alterations in adhesion activities could account for the matrix accumulation as seen in glaucoma patients [191]. Action of EC-derived EVs on annulus fibrosus (AF) cells causes the enhanced matrix catabolism, which consequently induce neo-angiogenesis in the degenerating disc. Likewise, the AF cells treated with EC-derived EVs induced the MMP activity by increasing the expression of MMP-1, MMP-3 and MMP-13 at mRNA level and at protein level [192]. The response of these target cells is regulated by their microenvironment, which could be modified by MSC-produced MMPs and TIMPs. Such trophic activities of MSCs are actively being recognized for repairing and regeneration of osteochondral and other musculoskeletal tissues, such as tendon/ligament and meniscus [193].

EVs derived from non-pigmented ciliary epithelium were incubated with TM cells in dose dependent manner, which significantly decreased the expression of β-Catenin, GSK-3β in TM cells. Pro-MMP9 and MMP9 activities were significantly enhanced in TM cells treated with high concentrations of EV, indicating that these EVs modulate biological activities in recipient cells and regulate key canonical Wnt proteins expression in the eye model for the human ocular drainage system [194]. Likewise, the treatment of MSC-derived EVs to corneal stromal cells (CSCs) induces the upregulation of MMPs and downregulation of ECM-related proteins such as collagens and fibronectin in CSCs. The findings indicate that adipose-MSCs might play an important role in the regulation of CSC viability through ECM remodeling, via EVs [195].

Moreover, EVs secreted by corneal fibroblasts can transport MMP-14 to vascular ECs, as a prerequisite step for the recruitment/packaging of MMP-2 into corneal fibroblast-derived EVs [43]. Such transport mechanism governs the MMP-14 activity in corneal angiogenesis and could serve as an important component for targeting angiogenic processes in the cornea. This is in agreement with a recent study which shows that MMP-14 modulates signal transduction and angiogenesis in the cornea [196].

### 9.3. Cardiac Repair

It has been shown that ischemic EVs confer protection against oxidative-induced lesion, promote proliferation and sprouting of ECs and stimulate cardiac angiogenesis [197]. Ischemic EVs display higher levels of MMPs and promote the secretion of MMP by ECs. The angiogenic effect of ischemic EVs is partially recapitulated through EV-mediated transport of miR-222 and miR-143. Additionally, intramyocardial delivery of ischemic EVs improves neovascularization following MI [197]. It is believed that the excessive myocardial fibrosis is the main pathological process in the development of cardiac damage and heart failure. During mechanical stress, the myocardial fibrosis is significantly increased and fibroblast hyperplasia is induced, as well as the induction of collagen and MMPs expression both in vivo and in vitro. However, there is a repair mechanism against mechanical stress, as shown by miRNA-378 secretion from cardiomyocytes and its transport via EVs, which acts as inhibitor of excessive myocardial fibrosis during early stage of cardiac hypertrophy [198]. Additionally, EVs released by cardiac TCs are transferred to the damaged myocardium and play active role in normal heart physiology and regeneration [199].

Additionally, TCs have been reported to secrete EVs into interstitial environment, which serve as mediators of long-distance paracrine activities of the TCs in adult organs [8,9,199,200,201,202]. As such, EVs release by cardiac TCs are considered essential component in the paracrine effects of TCs in heart, which are transferred to the damaged myocardium and contribute to normal heart physiology and regeneration [199].

### 9.4. Cutaneous Repair and Healing of Wounds

Regulation of EV-associated proteolytic activities have been shown to play active role in tissue homeostasis and wound healing processes through the recruitment of wound healing factors at the site of injury [111,203]. EVs play active roles in cutaneous repair by matrix remodeling. For instance, intravenous injection of adMSC-EVs promotes ECM reconstruction in cutaneous wound repair by regulating fibroblast differentiation to alleviate scar formation, and by regulating the ratios of collagen type III: type I, TGF-β3:TGF-β1 and MMP-3:TIMP [204]. Likewise, human induced pluripotent stem cell-derived EVs ameliorate the aging of human dermal fibroblasts (HDFs) by regulating the expression of matrix-degrading enzymes MMP-1/3, and collagen type I in HDFs [205].

Mesoangioblasts are vessel-associated progenitor stem cells endowed with the ability of multipotent mesoderm differentiation and represent a promising tool in the regeneration of injured tissues. EVs released by mesoangioblasts contain Hsp70, which interact with Toll-like receptor 4 and CD91, and regulates MMP-2 and MMP-9 expression to stimulate cell migration. Therefore, mesoangioblast-derived EVs regulate mesoangioblast stem cells’ ability to traverse the ECM [206].

Platelet-derived EVs could stimulate the production of MMPs in human umbilical vein ECs, which favor the angiogenesis [207]. Additionally, AdMSC-EVs induce the expression of type I collagen in vaginal fibroblasts from women with stress urinary incontinence (SUI). Treatment of fibroblasts with adMSC-EVs upregulates the expression levels of TIMP-1 and TIMP-3 in fibroblasts, with significant downregulation of MMP-1 and MMP-2 expression levels [208]. Therefore, adMSC-EVs may have therapeutic application for treating SUI by increasing collagen synthesis and decreasing collagen degradation in vaginal fibroblasts.

Additionally, BM-MSCs secrete hyaluronan-coated EVs, which carry CD44 mRNA. The hyaluronan coat on EVs may regulate their interactions with target cells and participate in ECM remodeling. The secretion of hyaluronan-coated EVs by MSCs is a general process, which contributes to several of the mechanisms of hyaluronan-mediated tissue regeneration [105]. It was shown that EMT enhances the secretion of EVs that carry CD44 and hyaluronan, which act as regulators in EV interactions with their targets and ECM remodeling during tissue repair [106]. EVs produced by local fibroblasts in the Duchenne muscular dystrophy patients are able to induce phenotypic conversion of normal fibroblasts to myofibroblasts, thereby increasing the fibrotic response [209]. This conversion is related to transfer of high levels of miR-199a-5p and to reduction of its target caveolin-1; both, therefore, are potential therapeutic targets in muscle fibrosis. It is tempting to appreciate that secretome of pericytes including ECM components and EVs is considered a rich source for tissue regeneration [210]. In summary, EV-associated matrix remodeling proteins hold therapeutic potential for tissue regeneration and amelioration of organ functions.

## 10. Concluding Remarks

The participation of EVs in the development of various diseases and tissue repair has well been elaborated for their nucleic acid and proteomic contents. However, the secretion and biological activities of EV-associated matrix-remodeling enzymes and their regulators are only just beginning to emerge. Nevertheless, there is still limited knowledge on the topological localization of ECM-degrading enzymes in relation to EVs, and futures studies are needed to answer several questions: if the enzymes are soluble or membrane bound; if the membrane bound-enzymes are membrane integral proteins or peripheral proteins; if the peripheral proteins are localized on the inner surface of EVs or on the outer surface; if the catalytic site of the integral proteins are inside the EVs or outside of EVs. This will help better understanding of matrix biology, and tissue remodeling during the progression of several human diseases reviewed in this article such as tumor, cardiovascular and arthritic diseases and could help developing new strategies for tissue repair. However, the available knowledge for understanding the role of EV-mediated matrix remodeling in neurological diseases, metabolic diseases and infectious diseases is scarce in the literature.

**Figure 2:**
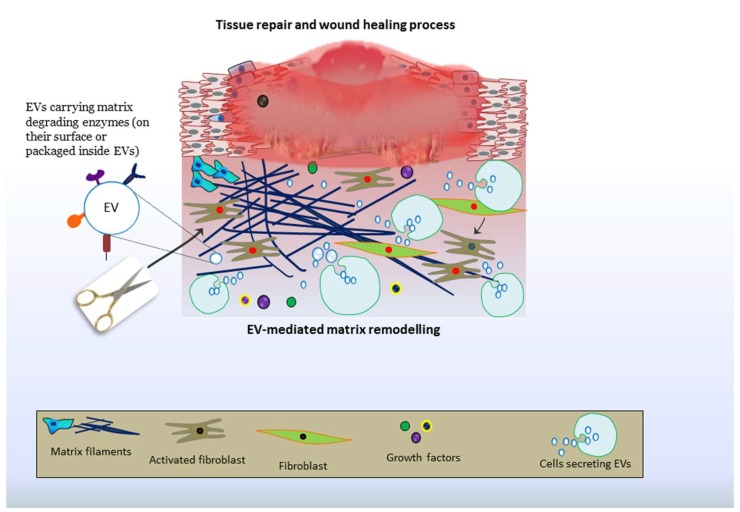
Schematic illustration for the roles of EV-associated MMPs in tissue repair: EV-associated matrix modeling enzymes stimulate the repair processes through matrix remodeling.

Finally, the content and concentrations of MMPs in patient-derived EVs could help identifying complimentary biomarkers and may have prognostic values. The most pivotal aspect of EV-mediated matrix remodeling could be harvested for tissue repair and ameliorating organ functions (Figure 2). As such, based on current knowledge, EVs can be tailored for tissue engineering and could serve a potential source of regenerative medicine aimed at organ therapies, particularly cardiac therapy, joint repair and musculoskeletal therapies.

## Figures and Tables

**Table 1:** List of matrix modulating enzymes and their regulators detected in extracellular vesicles.

MMPs and MMP Inducers or Inhibitors in EVs	Proteolytic Activities	Reference
**MMP-14 (MT1-MMP)**	Proteolytic activities in extracellular environment. Activation of pro-MMP2. Metastasis and angiogenesis in lung and ovarian cancer. Breakdown of capillary membrane. Proteolytically active mediators of matrix destruction in smokers	[32,42,43,53,78,79]
**EMMPRIN**	Induces the production of MMPs in recipient cells, induce tumor angiogenesis and favors the tumor progression	[54,80,81,82,83,84]
**MMP-2, MMP-9**	Immunosuppression in seminal vesicles during reproduction processes. Degradation of gelatin. Proangiogenic activity of PDGF-EVs Breakdown of capillary membrane. Cancer progression	[58,79,85,86,87,88,89,90,91,92]
**MMP-13**	ECM degradation and promote arthritis. Protects cartilage bone from degradation in osteoarthritis. Metastasis of nasopharyngeal cancer	[93,94,95,96,97]
**MMP-1, MMP-3, MMP-13**	Angiogenesis and tumor proliferation and invasion in prostate cancer. Pathogenesis of osteoarthritis and communication between joint tissue cells	[93,98]
**ADAM17**	Pathogenesis of human abdominal aortic aneurysm in smokers. Substrate shedding on distant cells	[99,100]
**ADAM10**	Enhanced cell motility, endothelial cell migration and VEGF-associated angiogenesis in cancer-associated fibroblasts. Sorting of CD23 protein into EVs which mediates IgE production and allergic response. Abdominal aortic aneurysm in smokers	[99,101]
**ADAM15**	Tumor suppressive activities by repressing cell adhesion, growth and migration	[102]
**ADAMTS-5, TIMP-3**	Aggrecan degradation and progression of arthritis, brevican degradation and invasion of glioma cells and rheumatoid synovial fibroblasts	[103]
**TIMP-1**	Induction of pro-tumorigenic and angiogenic processes during lung cancer progression	[104]
**Hyaluronan**	Hyaluronan-mediated tissue regeneration	[105,106]
